# Supplementary material for: Platelet Microparticles Protect Acute Myelogenous Leukemia Cells against Daunorubicin-Induced Apoptosis
Source: Cancers (Basel). 2021 Apr 14;13(8):1870. doi: 10.3390/cancers13081870 (PMC8070730; doi:10.3390/cancers13081870)

cancers

Supplementary Materials

Platelet Microparticles Protect Acute Myelogenous Leukemia Cells against Daunorubicin-Induced Apoptosis

Daniel Cacic, Håkon Reikvam, Oddmund Nordgård, Peter Meyer and Tor Hervig

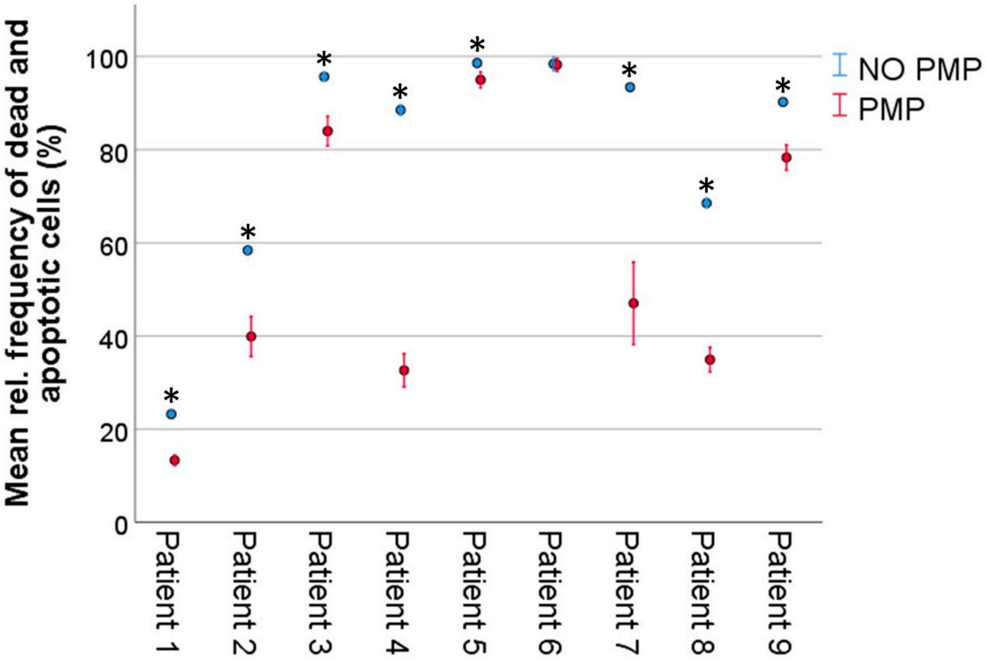

Figure S1. Resistance to daunorubicin (DNR)-induced cell death in primary acute myelogenous leukemia (AML) cells, individual data. Primary AML cells were co-incubated with PMPs for 24 hours and treated with 0.5 µM daunorubicin for another 24 hours before analysis ($n = 4$). Frequency of dead and apoptotic cells in individual patient samples was compared with or without co-incubation with PMPs using the paired-sample t test. * $p < 0.05$.

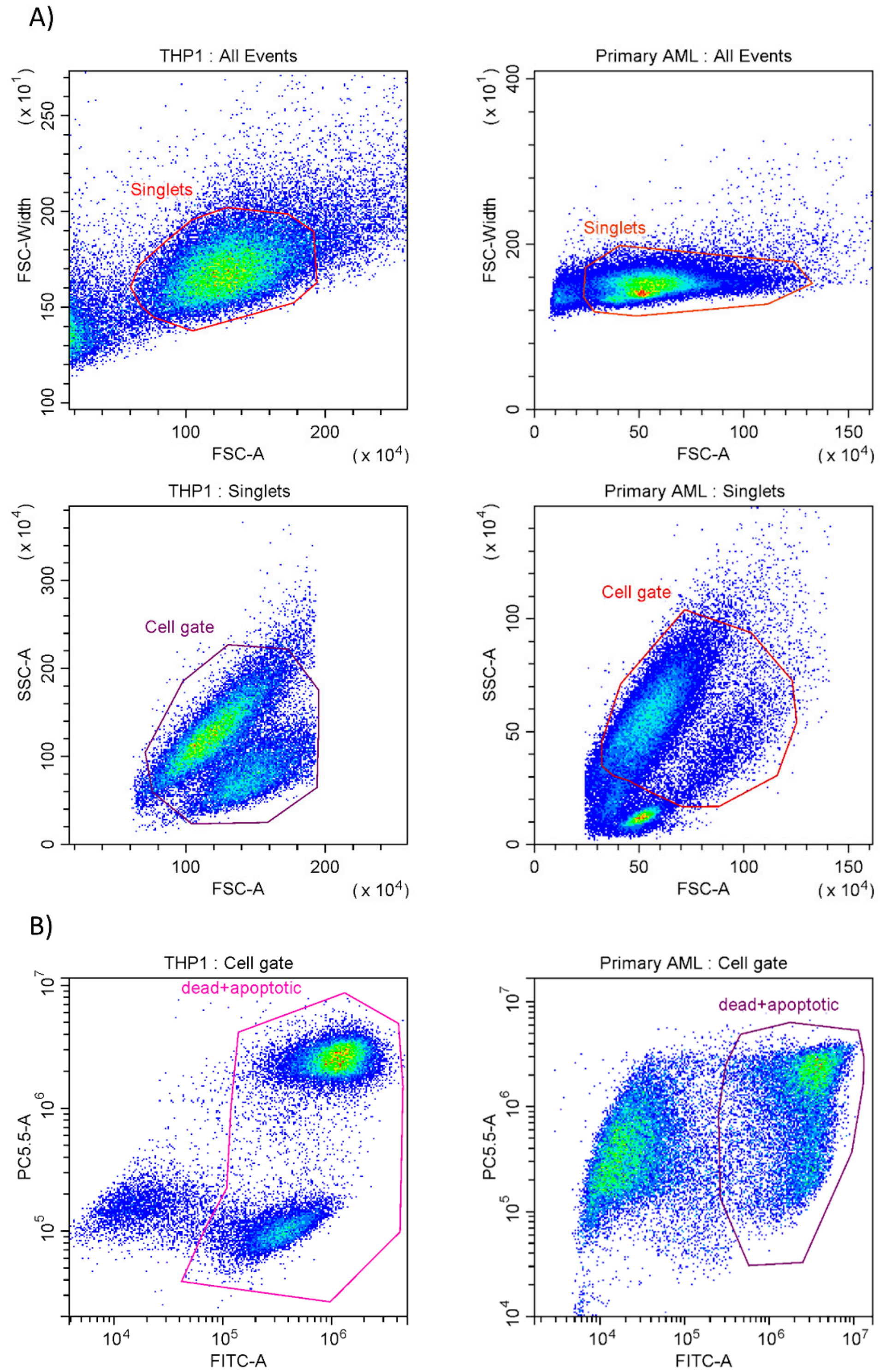

Figure S2. Gating strategy for flow cytometric apoptosis assay. (**A**) Doublets were discriminated using FSC-A versus FSC-width plots and "Cell gate" was set using FSC-A versus SSC-A plots in the singlets population. (**B**) Example of gating of dead and apoptotic cells in THP-1 and primary AML.

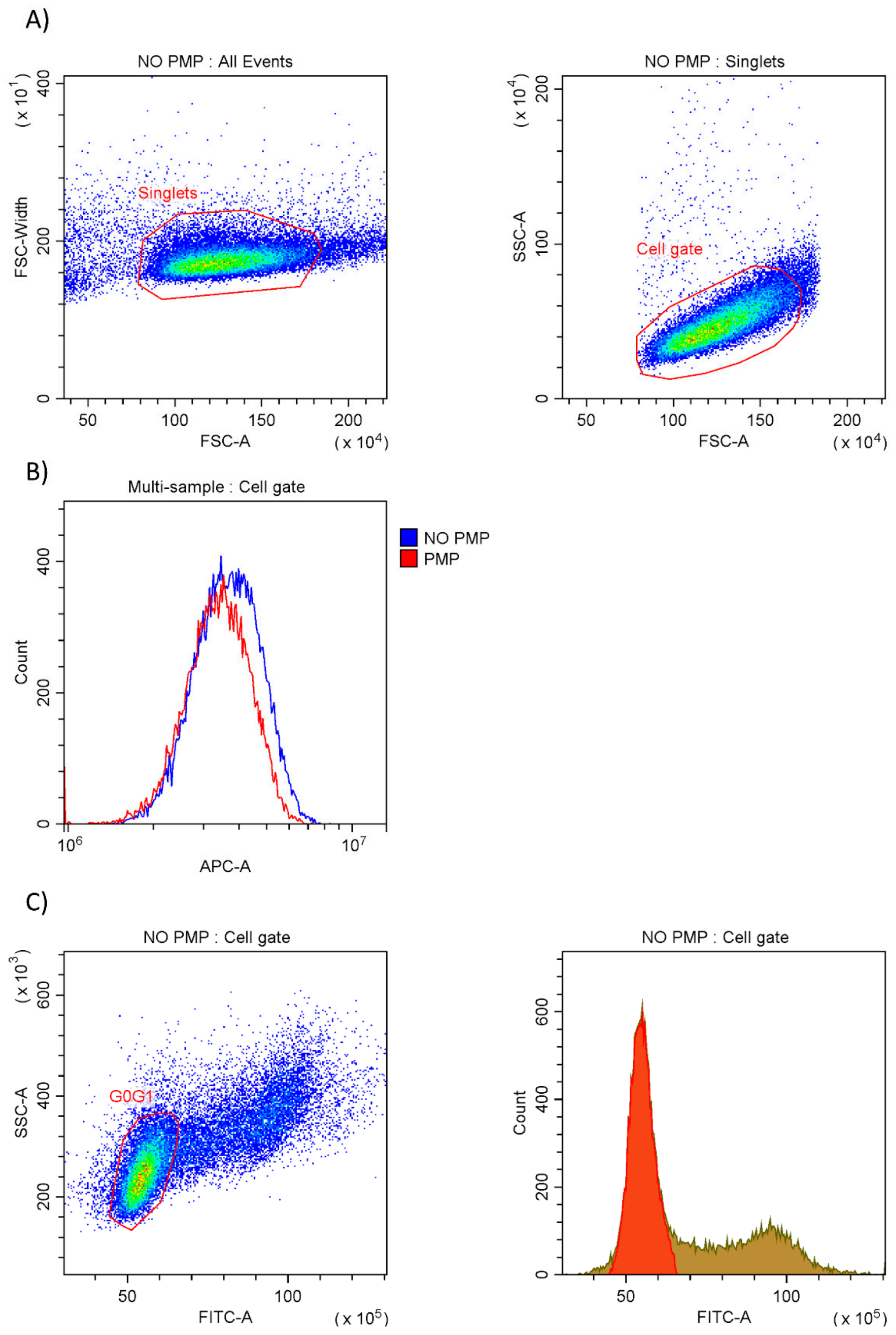

Figure S3. Gating strategy for flow cytometric analyses. (**A**) Doublets were discriminated using FSC-A versus FSC-width plots and "Cell gate" was set using FSC-A versus SSC-A plots in the singlets population, including only the low side scatter population. The plots are representative for "Cell gate" for caspase-9, proliferation, cell cycle, mitochondrial membrane potential, and intracellular protein analysis. (**B**) Histogram from representative experiment of mitochondrial membrane potential analysis. **C**, Pseudo color plot and histogram from representative experiment of cell cycle analysis.

Table S1. TaqMan assays used in this study.

Gene	Assay ID	miR	Assay ID
ACTB	Hs99999903_m1	hsa-miR-15a-5p	000389
BCR	Hs01036532_m1	hsa-miR-20b-3p	002311
CDK4	Hs00364847_m1	hsa-miR-26a-5p	000404
		hsa-miR-125a-5p	002198
		hsa-miR-125b-5p	000449
		hsa-miR-199a-5p	000498
		hsa-miR-223-3p	002295
		mmu-miR-451	001141
		hsa-miR-5683	475360_mat

Table S2. Description of antibodies used in this study.

Primary Antibody	Host	Clone	Supplier	Dilution	Incubation	Incubation Temperature
CDK4	Mouse	DCS-31	Thermo Fisher	1:40	60 min	Room temp.

Secondary Antibody	Host	Species Reactivity	Conjugate	Supplier	Dilution	Incubation	Incubation Temperature
Goat anti-Mouse IgG (H+L)	Goat	Mouse	DyLight 488	Thermo Fisher	1:25	30 min	Room temp.

Supplementary Methods

The following procedure was performed on images in JPG-file format at a resolution of 4640x3472 pixels.

1. Adjust brightness (210)
2. Subtract background (50 pics)
3. Enhance contrast (1.0%)
4. Gaussian Blur (2.0 pics)
5. 8 bit conversion
6. Threshold (auto)
7. Close
8. Fill holes
9. Close persistent holes manually with paintbrush tool, cross check with original image to verify correct outline.
10. Fill holes
11. Remove dark outliers (20.0 pics).
12. Remove obvious debris, clusters of cells, and cells cut on the edge of the image.
13. Analyze particles

Values will have to be adjusted depending on the quality and resolution of the original image when applying this protocol on other data sets. A representative analysis with raw and processed images are supplied below:

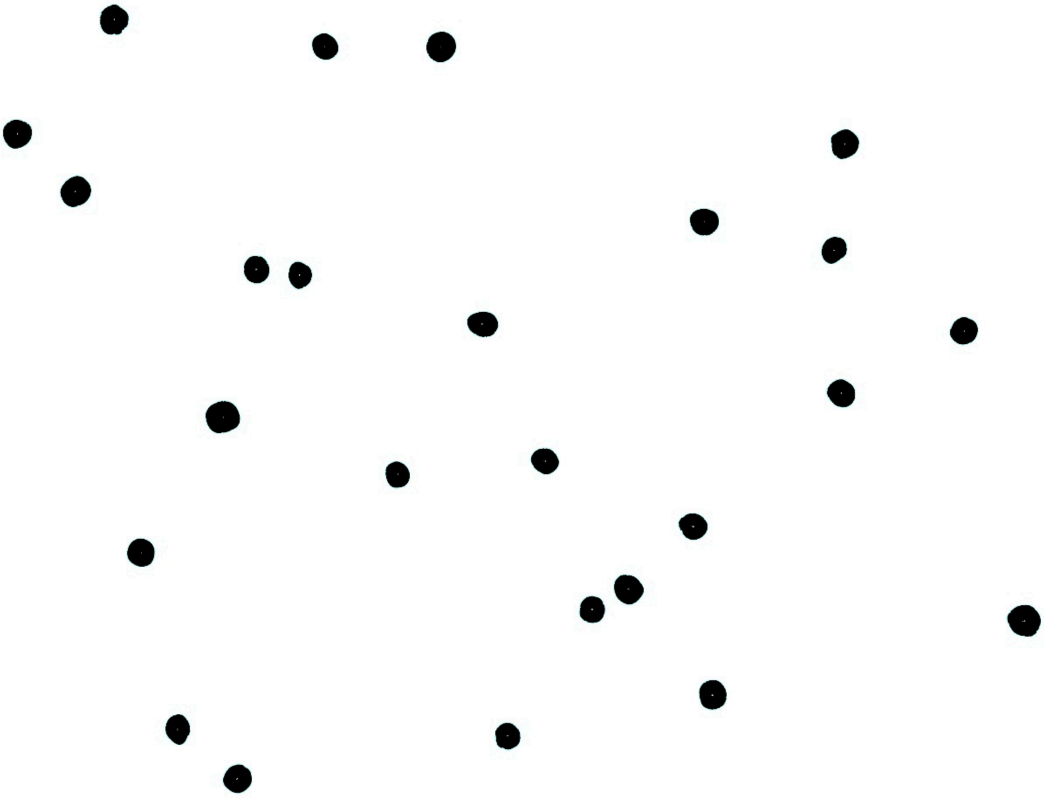

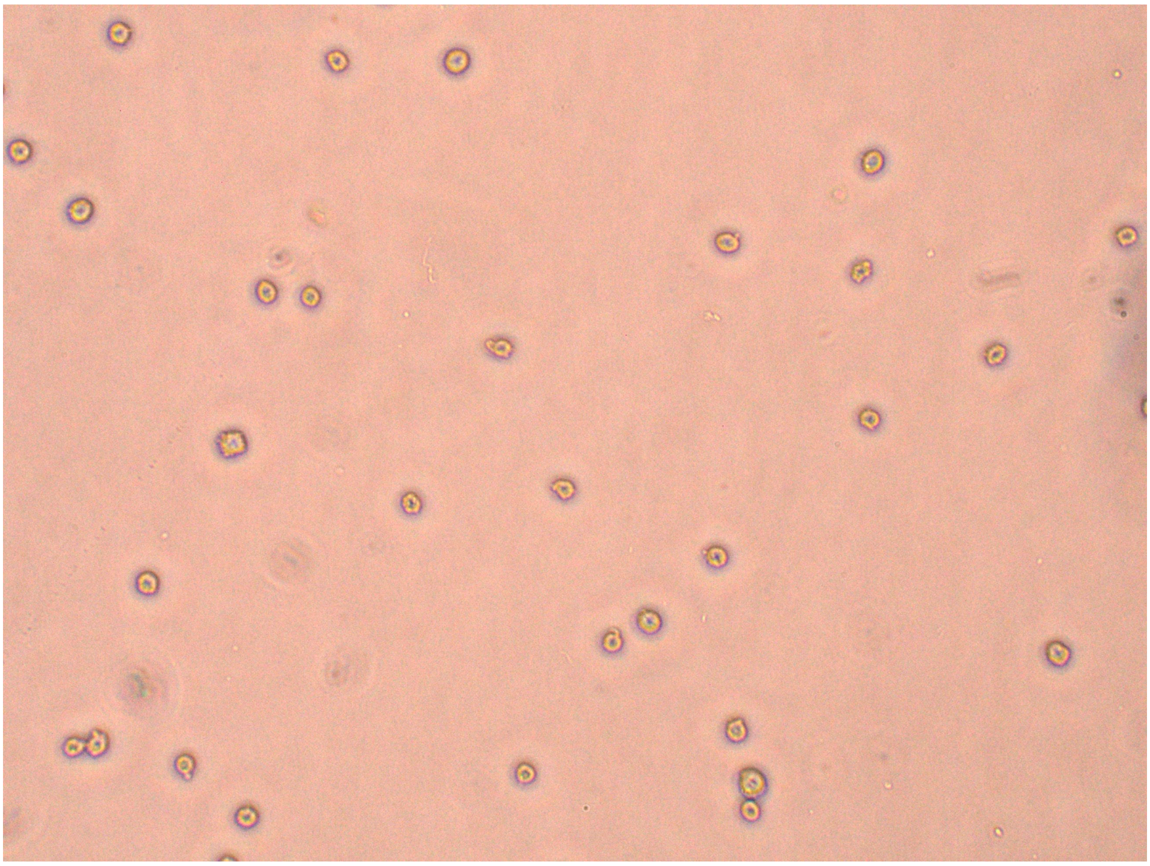

Supplement: Supplementary file 1 [file cancers-13-01870-s001.pdf]
